# Temperature-dependent mitochondrial-nuclear epistasis

**DOI:** 10.17912/micropub.biology.000147

**Published:** 2019-09-09

**Authors:** Enrique Cazares-Navarro, Joseph A Ross

**Affiliations:** 1 Department of Biology, California State University, Fresno, CA USA 93740

**Figure 1.**
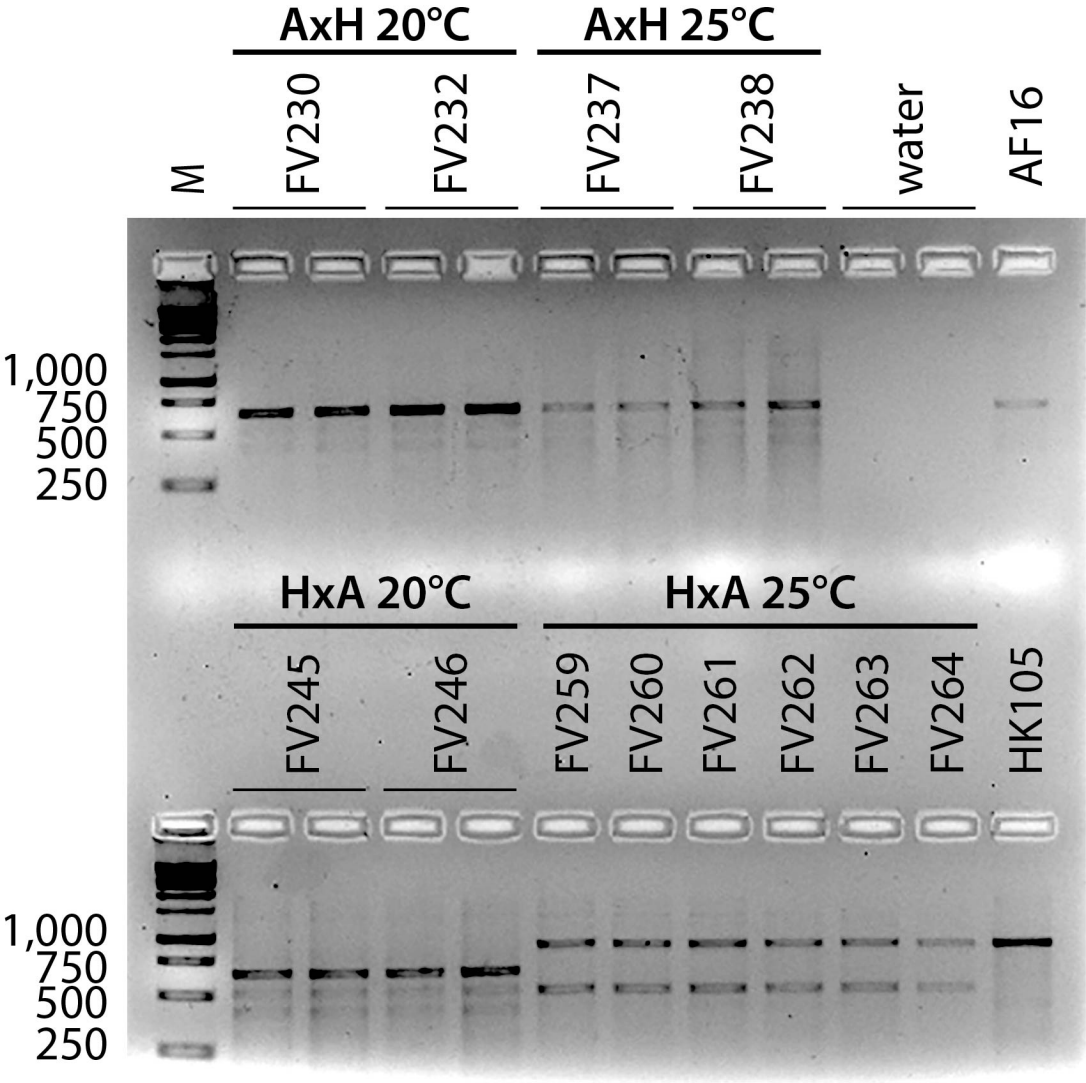
Agarose gel electrophoresis genotype analysis of some of the AF16-HK105 AI-RIL at the cb-m127 indel marker. Six AI-RIL genomic DNA templates (FV###) and control templates (positive: AF16 and HK105 genomic DNA samples; negative: water) were PCR amplified and then electrophoresed. For quality control purposes, the other six AI-RIL templates shown were amplified in duplicate. The AF16 control amplicon is ~600 bp in size; the HK105 control amplicon is ~900 bp in size, as predicted by (Koboldt *et al.* 2010). M: 1 kb ladder (Promega, G5711).

## Description

The mitochondrial and nuclear genomes contain genes encoding the protein subunits comprising mitochondrial electron transport chain complexes I–V (Blanchard and Lynch 2000). This dynamic has established a co-evolutionary relationship between the two genomes: when a deleterious mutation occurs in one genome, a compensatory mutation in the other genome might restore efficient oxidative phosphorylation (Blier *et al*. 2001). The epistatic interactions between different co-evolved mito-nuclear alleles can then be disrupted when mating between two genetically diverse populations occurs (Rand *et al*. 2004). Such hybridization introduces non-compensatory nuclear alleles that can again disrupt efficient electron transport (Burton *et al*. 2013). The nematode *Caenorhabditis briggsae* exhibits such evidence of mito-nuclear epistasis, where experimental recombination of the nuclear genome from the wild isolate AF16 with the mitochondrial genome of wild isolate HK104 (and *vice versa*) resulted in hybrid dysfunction (Chang *et al*. 2016). Further, AF16-HK104 advanced-intercross recombinant inbred lines (AI-RIL) show strong epistatic interactions between the mitochondrial and nuclear genomes, particularly with the X chromosome (Haddad *et al*. 2018). AF16 and HK104 are representatives of the genetically divergent tropical and temperate clades of *C. briggsae* (Cutter *et al*. 2006). Because of this phylogeographic population structure and knowing that mitochondrial genetic variation can play important roles in environmental adaptation (Das 2006, Camus *et al*. 2017, Lamb *et al*. 2018), we tested the hypothesis that mitochondrial-nuclear epistatic interactions in *C. briggsae* are temperature-dependent. We hybridized AF16 with a different temperate population, HK105. We created ten (10) replicate AF16-HK105 AI-RIL for both (2) cross directions at both (2) 20°C and 25°C. We then genotyped these forty (10x2x2) RIL at a single locus on the X chromosome to measure segregation distortion and its dependence on cross direction (and thus on cytotype and mitotype).

Samples of the wild isolate *C. briggsae* populations AF16 and HK105 were obtained from the Caenorhabditis Genetics Center. Nematodes were cultured on NGM plates with *Escherichia coli* strain OP50 as a food source according to (Steirnagle 2006). AF16-HK105 advanced-intercross recombinant inbred lines (AI-RIL) were produced as previously described (Ross *et al*. 2011) by crossing males from one population with a self sperm-depleted hermaphrodite from the other population to generate the F1 generation. Seven subsequent generations of sibling-mating and then ten generations of self-fertilization by single hermaphrodites were used to produce each AI-RIL. Ten biological replicate lines were established at the F1 generation for reciprocal crosses AF16xHK105 and HK105xAF16 (male population listed first). This cross design was performed both at 20°C and 25°C to produce lines FV225-234 (AF16xHK105 at 20°C), FV235-244 (AF16xHK105 at 25°C), FV245-254 (HK105xAF16 at 20°C) and FV255-264 (HK105xAF16 at 25°C). Genomic DNA was extracted from each line by phenol-chloroform extraction and isopropanol precipitation in preparation for PCR genotyping. Genomic DNA was diluted to 5 ng/µL in 10mM TRIS buffer pH 8.0. Amplification and agarose gel electrophoresis was performed according to (Chang *et al.* 2016). The cb-m127 insertion-deletion (indel)-amplifying primer sequences were obtained from (Koboldt *et al*. 2010), and the primers were synthesized by Integrated DNA Technologies (Coralville, IA, USA). The cb-m127 indel is X-linked (Haddad *et al*. 2018).

The genotype (either homozygous AF16, heterozygous, or homozygous HK105) of each of the forty AI-RIL was identified based on agarose gel electrophoresis banding pattern. The genotype distribution for each of the four sets of lines was compared to the neutral expectation using chi-square tests with an initial significance threshold of p =< 0.05. The null hypothesis for allele retention of hybrid lines in the absence of selection is that half of replicate line alleles will represent one P0 population allele and half the alleles from the other P0 generation population (in this case: five lines homozygous for AF16 and the other five lines homozygous for HK105). Analyses were Bonferroni-corrected for multiple tests (two cross directions * two temperatures = four tests) to a significance level of p < 0.0125.

In Figure 1, all AxH (AF16xHK105) samples, and the 20°C HxA (HK105xAF16) samples are homozygous AF16 at indel marker cb-m127; all 25°C HxA samples are homozygous HK105. Additional gels (not shown) revealed that all ten AxH 20°C and 25°C replicate lines retained the AF16 cb-m127 allele, as did all HxA 20°C lines. All ten HxA 25°C lines retained the HK104 cb-m127 allele. As expected for AI-RIL, no line was identified as heterozygous for the AF16 and HK105 alleles. Smaller, non-diagnostic amplicons of varying intensity are evident in amplifications of all of the AI-RIL and also much more faintly in the positive control templates. These smaller amplicons might result from a combination of suboptimal cycle sequence conditions for the primer pair used, generating amplicons from multiple loci. Differences in intensity between the diagnostic amplicons and the non-specific amplicons might also be due to differences in concentration between the template samples. No amplicons originate from a contaminating template, because the negative control lanes are blank.

Two striking patterns are evident in the AI-RIL genotypes. First, within each of the four sets of ten replicate lines, multiple genotypes were never observed. This result is statistically significantly different than the neutral expectation of half of the replicates retaining each parental allele. Second, the X allele inheritance pattern was observed to differ both by cross direction and also by temperature. At 25°C, the AxH lines retained only AF16 X alleles, but the HxA lines retained only HK105 X alleles. At 20°C, the HxA lines retained only AF16 X alleles, but at 25°C, lines of the same cross direction (HxA) retained only HK105 X alleles. These data reveal temperature-dependent mito-nuclear epistasis, in which cross direction and rearing temperature correlate with distinct inheritance patterns of X chromosome alleles in a hybrid nuclear genetic background. The X chromosome has been reported to be an ideal nuclear locus for alleles that are co-adapted to cytoplasmic factors (Rand *et al*. 2004, Rogell *et al*. 2014), so it is not surprising that we have identified strong mito-X linkage disequilibrium in these *C. briggsae* hybrids. These data contribute to a growing body of literature connecting environmental factors like temperature to epistatic interactions involving the mitochondrial genome (Arnqvist *et al*. 2010, Dowling *et al*. 2007, Paliwal *et al*. 2014).

## Reagents

Purified genomic DNA from all forty AI-RIL are available from the authors upon request. Some AI-RIL were not cryopreserved because of inadvertent desiccation. FV254–258 and FV260-265 are available from the authors upon request.
